# Combination of Dry Milling and Separation Processes with Anaerobic Digestion of Olive Mill Solid Waste: Methane Production and Energy Efficiency

**DOI:** 10.3390/molecules23123295

**Published:** 2018-12-12

**Authors:** Doha Elalami, Hélène Carrère, Karima Abdelouahdi, Abdallah Oukarroum, Driss Dhiba, Mohamed Arji, Abdellatif Barakat

**Affiliations:** 1 LBE, Université de Montpellier, INRA, 102 Avenue des Etangs, F-11100 Narbonne, France; Doha.ELALAMI@um6p.ma; 2 LCME, FST Marrakech, Université Cadi Ayyad, Marrakech 40000, Morocco; abdelouahdi@gmail.com; 3 Mohammed VI Polytechnic University (UM6P), 43150 Benguerir, Morocco; Abdallah.OUKARROUM@um6p.ma (A.O.); D.DHIBA@ocpgroup.ma (D.D.); 4 OCP Group, Complexe industriel Jorf Lasfar, BP 118 El Jadida, Morocco; mohamed.arji@ocpgroup.ma; 5 IATE, CIRAD, Montpellier SupAgro, INRA, Université de Montpellier, 34060 Montpellier, France; abdellatif.barakat@inra.fr

**Keywords:** anaerobic digestion, pretreatment, energy efficiency, electrostatic separation, milling, sieving

## Abstract

This experimental work aims at investigating the effects of milling; sieving; and electrostatic separation on the biochemical methane potential of two olive pomaces from traditional olive oil extraction (M) and from a three-phase system (T). Sieving proved to be efficient for increasing the soluble chemical oxygen demand in the smallest fractions of the sieve of both M (62%) and T (78%) samples. The positive fraction following electrostatic separation also enhanced chemical oxygen demand (COD) solubilisation by 94%, in comparison to sample T milled at 4 mm. Sieve fractions with a size greater than 0.9 mm contained 33% and 47% less lipids for the M and T biomasses; respectively. Dry fractionation modified sample properties as well as lipid and fiber distribution. Concomitantly; milling increased the accessibility and facilitated the release of organic matter. The energy balance was positive after knife milling and sieving; while ball milling and ultrafine milling proved to be inefficient.

## 1. Introduction

The production of olive oil was estimated at 2,539,000 tons worldwide in 2016 and the EU is the first producer attaining 69% of international production. In Tunisia and Morocco, the leading olive producers in North Africa, 1,850,000 and 1,000,000 ha of land, respectively, are planted with olive trees. In Morocco, 75% of olive production is intended for oil extraction. There are three types of processes for oil extraction: the traditional press consisting of grinding olives and transforming them into paste, the two-phase and three-phase processes involving washing the olives, milling them, and finally a centrifugation step using hot water for the three-phase process [1]. The oil recovery yield is the highest after the three-phase process, followed by the two-phase process and lastly by the traditional press.

In all cases, an olive mill solid waste (or olive pomace) is co-produced. Its quantity and quality depend on the extraction process, production, and agricultural conditions. This waste is mainly wet and contains stones, flesh and olive skin and is rich in fibers, in phenols and mineral compounds as well as certain toxic compounds. When olive mill waste is used as an amendment for soil, it supplies easily degradable organic matter to the soil [2]. However, it has negative effects on seed germination, plant growth, and microbial activity because of its low pH and phenols, tannins, and fatty acid content [1]. In addition, the accumulation and storage of olive pomace poses a serious environmental problem.

Due to its oil content, the burning of olive pomace as a source of heating energy is very common in Moroccan rural regions and the residues are applied to the soil. However, the combustion of oily olive waste favours the release of toxic compounds [3]. Indeed, several methods can be used to extract energy from olive pomace while reducing its harmful impacts on the environment. Hydrothermal carbonization can convert wet organic waste or biomass into coal-like solids, thus imitating the natural coalification process. It has already been applied to olive solid wastes to produce hydrochar, which is more carbonaceous than lignite with a similar higher heating value [4]. Yet, the residual liquid needs to be treated to reduce its potential effects on environment [5].

Besides thermochemical processes, anaerobic digestion produces energy through the biological conversion of organic matter into biogas. It has low nutrient and energy requirements, reduces waste volume and even stabilises it. Anaerobic digestion could therefore represent a suitable treatment for olive pomace. Indeed, the biochemical composition of olive pomace (OP) would favor a high theoretical methane production [6]. For example, Fezzani et al. reported that, on a dry basis, olive pomace contains about 35% carbohydrates, 44% lignin, 12% proteins, and 10% lipids [7]. The lignin and polyphenol content of OP causes its biodegradation to be difficult; for this reason, a preliminary pretreatment would be needed to enhance methane production from OP.

The effects of pretreatments such as thermal pretreatment have previously been investigated. Results demonstrate improvement in the bioavailability of olive pomace cobalt [8], as well as an increase the solubilization of the chemical oxygen demand. However, no effect on methane production was observed after a thermal treatment at 134 °C [9]. In addition, ultrasound pretreatment increased the methane yield by 13% while consuming less energy, i.e., 153 kJ/L compared to the 503 kJ/L needed for thermal pretreatment [10]. Besides thermal processes, chemical pretreatments of olive pomace biomass are also currently being applied. In some cases, these pretreatments utilize large quantities of water and chemicals and generate a significant amount of waste (e.g., effluents) [11]. Low or no water consumption during lignocellulosic pretreatment could decrease the generated effluents, and would also reduce the energy input for pretreatment of the biomass [12,13,14]. For this purpose, dry biorefinery processes requiring low water and chemical consumption and thus eliminating waste production have been proposed in recent years [12,14]. They include mechanical biomass size reduction (milling) and biomass fractionation processes such as sieving and electrostatic separation.

Electrostatic separation is the combination of two steps: the particles are first charged, then transferred to an electric field where they are separated according to their charge [15]. Electrostatic separation has previously been applied to wheat bran [16], oil cakes [17], and rice straw [18], although it has yet to be combined with anaerobic digestion. In a study on 3 mm screen size milling before anaerobic digestion of OP, the methane maximum production rate was enhanced, although no impact was observed on the methane yield [19]; in addition, the energy requirements in this study were not addressed. More generally, literature concerning the comparison of energy consumption and energy efficiency of chemical, physicochemical, and mechanical treatment of OP biomass still remains scarce.

The present work is a first attempt to compare different milling processes (i.e., knife milling, ball milling, or ultrafine milling), and sieving and electrostatic separation processes, in terms of biochemical methane potential and energy consumption. The changes in physicochemical properties, anaerobic digestion, energy consumption, and energy efficiency were assessed and compared to the chemical and physicochemical pretreatment methods developed in the literature for OP biomass.

## 2. Results and Discussion

**Figure 1:**
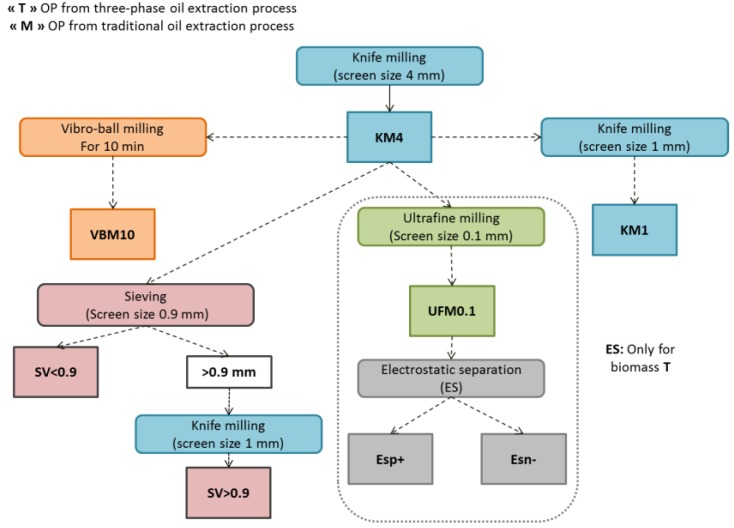
Dry fractionation process of olive pomace biomass developed in this study.

Two olive pomaces originating from different traditional oil extraction process (sample M) and from three-phase extraction process (sample T) were submitted to successive pretreatment and fractionation processes, as indicated in Figure 1. KM4, KM1, VBM10, and UFM0.1 samples were obtained after OP milling with different devices.

**Figure 2:**
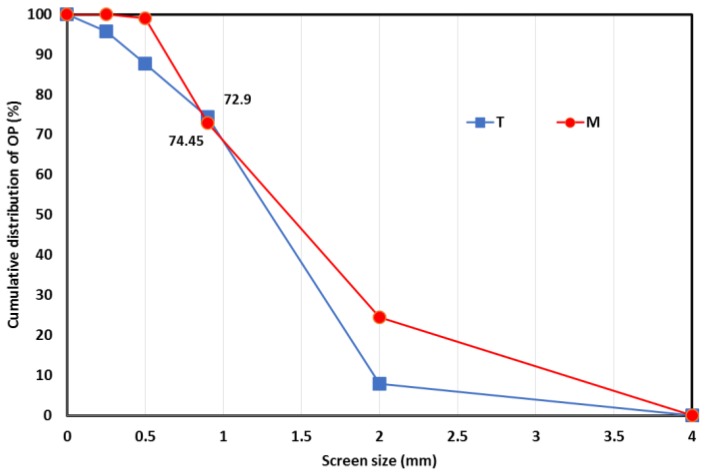
Olive pomace distribution after sieving.

With the objective of producing more effective fractions with increased methane production, OP samples were subjected to different dry separation processes, which would make them more easily accessible to microorganisms. The sieving process is a basic mechanical separation of particles according to shapes and sizes. First, the size distribution of OP was characterized by successive sieving of KM4 fractions using different screens, as illustrated in Figure 2. As the largest fraction was separated by a 0.9 mm screen, the latter was used for the sieving fractionation process and two OP fractions were produced: SV < 0.9 (fraction with a particle size lower than 0.9 mm) and SV > 0.9 (fraction with a particle size higher than 0.9 mm). The SV > 0.9 fractions accounted for 72.9% and 74% of the entire sample for biomasses M and T, respectively. Finally, a pilot electrostatic separator was also applied to olive pomace T: Fractions ESp+ (positively charged fraction) and ESn− (negatively charged fraction) were thus obtained from UFM0.1 (Figure 1).

### 2.1. Effects of Dry Fractionation on OP Biochemical Composition

OP fractions obtained after fine milling and after separation processes by sieving and electrostatic separation were characterized in terms of biochemical composition and physicochemical proprieties. Table 1 indicates that after separation, samples present different contents in sugars, proteins, and lipids compared to VBM10. In particular, both M and T olive pomaces had similar VS and TS contents. However, their biochemical compositions were different. The T sample contained 16% hemicellulose, 10% cellulose and 31% lignin, compared to 11% hemicellulose, 11% cellulose, and 37% lignin in the M sample. For both varieties, proteins represented around 5% of the total weight of VBM10. However, the lipid content of M was around 14%, while only 6% lipids were measured in T samples. This difference is due to the oil extraction process: the three-phase process (sample T) is more efficient in extracting oil than the traditional technique (sample M).

Table 1 indicates the results of a triplicate analysis of the SV < 0.9 and SV > 0.9 fractions of M and T samples. There were significant differences in the biochemical composition of the SV < 0.9 and SV > 0.9 fractions obtained after sieving. In particular, SV > 0.9 fractions from M samples contained more fibers (27%) compared to VBM10 (22%) and SV < 0.9 (9%), as shown in Table 1. In contrast, SV < 0.9 fractions were mainly composed of pulp with a high lipid and protein content compared to VBM10. This result is consistent with the literature [20,21]. The SV > 0.9 fraction of M biomass contained about 13.11% hemicelluloses and 14.17% cellulose compared to only 4.16% and 4.99% of hemicelluloses and cellulose, respectively, in the SV < 0.9 fractions. In addition, the content of lignin was lower than in the SV < 0.9 fraction (Table 1). In contrast, for the T sample, a similar lignin content was observed in both fractions, while a lower content of hemicelluloses and cellulose was found in the SV < 0.9 fraction. Moreover, SV < 0.9 fractions from M and T samples had higher lipid contents: 30 and 6.2% compared to only 9.2 and 3.3% in the SV > 0.9 for M and T samples respectively. Similarly, the protein contents of the SV < 0.9 fractions were 9.20 and 10.87%, while they only reached 1.6 and 3.1% in the SV > 0.9 fractions of M and T samples, respectively. This difference clearly suggests that the sieving process might lead to the isolation of fibrous (rich in carbohydrates) and non-fibrous (rich in pulp, lipids, and proteins) fractions from the biomass material.

A pilot electrostatic separator was also employed for the production of two fractions displaying different compositions: ESp+ and ESn− from the T biomass. Table 1 summarizes the recovery yields and the characteristics of both fractions produced with ultrafine milling combined with electrostatic separation (UFM-ES. Results clearly indicate that ES has a significant influence on the biochemical and physicochemical proprieties of the OP fractions. The negatively charged fraction ESn− was characterized by finer particles (D_50_ = 49 µm) than the positively charged fraction ESp+ (D_50_ = 75 µm), whereas the starting material (UFM0.1) presented a D_50_ of 54 µm.

ES technology also influenced the color of the OP fractions. Color is useful for revealing the biomass separation and fractionation and has already been utilized for evaluating ES efficiency [20]. Colorimetric parameters correspond to luminosity (L*), green/red (a*) and blue/yellow (b*) balances. In agreement with previous studies, colorimetric analysis of UFM0.1, ESp+ and ESn− highlighted the efficiency of ES technology on OP fractionation and separation (Table 1). Indeed, Basset et al. [16], Hemery et al. [17], and Chuetor et al. [18] obtained similar results with wheat bran, oil cakes, and rice straw, respectively, after two successive ES. The negatively charged fraction ESn− was brownish (high L* and high b*), whereas the ESp+ fraction was almost black (low L* and low b*), while the starting material UFM0.1 was obviously a mixture of both fractions (Table 1). In the same manner, sieving affected color parameters. The SV < 0.9 fractions for both M and T samples were darker and greener than the milled samples, while the SV > 0.9 fractions were lighter and redder.

The ES fractionation also affected the biochemical composition of the positively ESp+ and negatively ESn− charged fractions, as displayed in Table 1. In agreement with Wang et al. [22], the results clearly highlight the potential of ES for carbohydrate, lignin, lipid, and protein separation from biomass under dry conditions without water or external heating. Indeed, the positively charged fraction ESp+ contained 8.9% of proteins and 41% of lignin while 3.4% proteins and 36% lignin composed the starting material UFM0.1 (Table 1). Negatively charged fraction ESn− was more concentrated in hemicelluloses (20%) and cellulose (15%) in comparison with 11.1% hemicellulose and 8.9% cellulose in ESp+ (Table 1).

To conclude, according to these results, the positively charged fraction ESp+ (black) contains heterogeneous and non-fibrous long particles (pulp). Conversely, the negatively charged fraction ESn− (brown) contains a greater amount of homogeneous and fibrous small particles. Basset et al. and Barakat and Mayer attributed the differences in color and morphology to the differences in biochemical composition, which in turn depend upon the origin of the plant tissues [16,23].

### 2.2. Effects of Milling and Dry Fractionation Processes on Methane Production

Grinding is expected to increase the surface area of OPs and to favor the release of organic compounds or COD into the liquid phase. While COD solubilization is generally associated with an increase in methane production, the release of high amounts of phenolic compounds may inhibit anaerobic digestion [24]. Firstly, to investigate the impact of mechanical pretreatments and dry separation on the release of organic matter during BMP tests, COD and polyphenol solubilization were measured for each sample. As demonstrated in Table 2, the KM4 of M and T presented the same order of magnitude of soluble COD content. However, the soluble polyphenol content in the KM4 of olive pomace T was 43% less than in the KM4 of M. Even though knife milling at 1 mm (KM1) did not lead to a significant variation in soluble COD nor soluble polyphenols in comparison to knife milling at 4 mm (KM4), increases in soluble COD of 74% and 40% were, respectively, observed after VBM-10 min of M and T samples. In addition, ultrafine milling of the T sample increased the soluble COD content from 115 mg/gVS (KM4) to 197 mg/gVS (UMF0.1). Similarly, soluble polyphenols increased from 4.2 to 12 mg/gVS. Variations in the soluble COD and polyphenol contents were also observed after sieving and electrostatic separation processes (Table 2). Subsequent to sieving, SV < 0.9 fraction had a higher soluble COD and polyphenol content for both OP samples. Soluble COD originating from the SV < 0.9 fraction of both M and T olive pomaces were 245 and 205 mg/gVS, respectively. Polyphenols solubilized from the SV < 0.9 fractions represented 10.6 and 7.5 mg EAG/gVS respectively for M and T olive pomaces compared to 7.4 and 4.2 mg EAG/gVS, respectively for both biomasses. After electrostatic separation, the positively charged fraction ESp+ was 22% more concentrated in soluble COD and polyphenols than the negatively charged fraction ESn− fraction. The maximum amount of soluble polyphenols observed in this work was 13.8 mg GAE/gVS in the positive fraction, corresponding to a concentration of 69 mg/L in the liquid fraction of anaerobic digestion tests. At this concentration, no inhibition was observed, in agreement with Hernandez et al. [25], who reported polyphenol inhibiting concentrations ranging from 800 to 1600 mg organic carbon per liter. In addition, another study has stated that for less than 1.5 g/L of phenolic compounds, methanogenic activity was not affected, and thus that methane production did not decrease [24].

**Figure 3:**
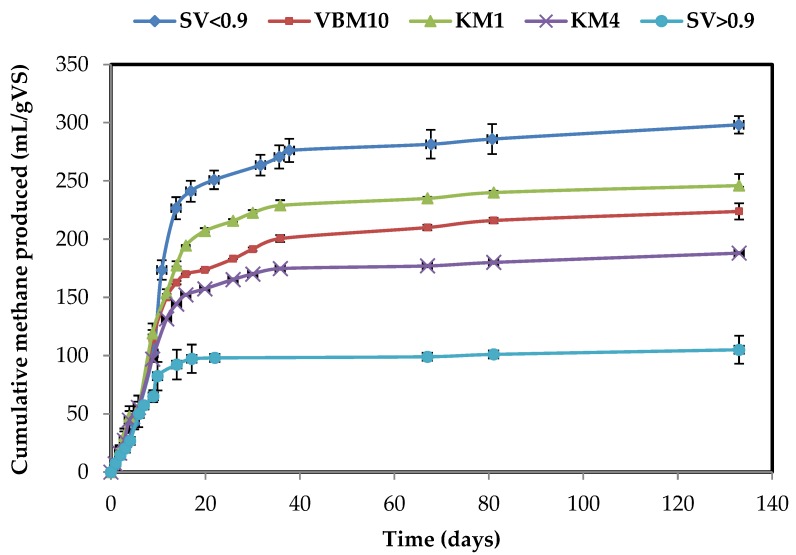
Cumulative methane production for different dry fractionation process of M olive pomace from traditional extraction process.

**Figure 4:**
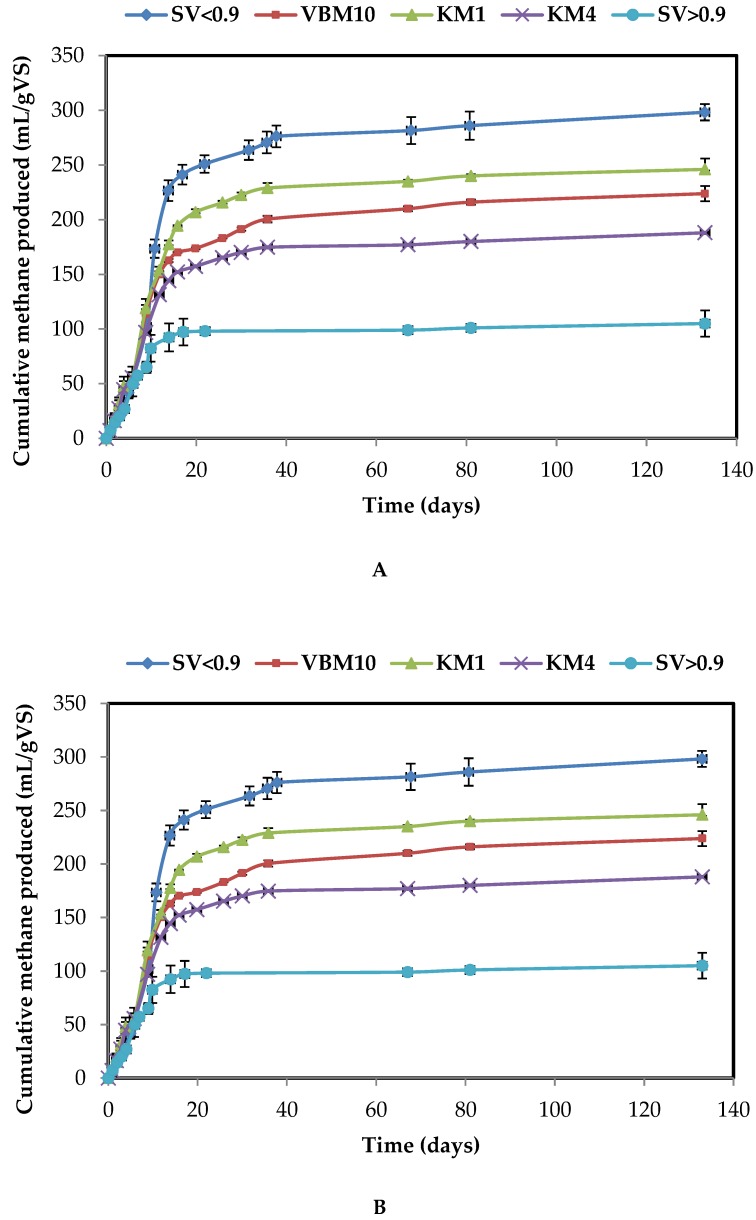
Cumulative methane production for different dry fractionation process of T olive pomace from three-phase extraction process: (**A**) Milling pretreatments and (**B**) dry separation process (electrostatic and sieving).

The biomethane potentials originating from OP after different mechanical milling and separation processes were further investigated (Table 2, Figure 3 and Figure 4).

Considering the 4 mm ground samples (KM4), the methane potential of the M sample (188 mL/gVS) was higher than for the T sample (98 mL/gVS). This can be explained by the higher lipid content in the M biomass compared to the T biomass. The theoretical methane yields from M and T olive pomace biomasses were 249 and 191 mL/gVS, respectively. Furthermore, the carbohydrate contents were 23 and 28% for M and T samples, respectively, whereas the protein contents were almost similar. Thus, the difference in methane production yields could be explained by the difference in lipid contents for the M and T biomasses (Table 1). BMP values of KM4 sample represent 76% and 58% of the theoretical values of M and T olive pomaces, respectively. However, after knife milling, the EMY/TMY ratio was enhanced. In addition, a comparison of the fractions obtained by sieving reveals that the smallest fraction (SV < 0.9) presents a higher ratio for both M and T samples (Table 2).

The methane production from M samples was found to be almost stabilized after the first 35 days. However, for T samples, the plateau in methane production was only reached after 130 days (Figure 3 and Figure 4).

Milling using 1 mm screen size (KM1) led to an enhanced methane production for both biomasses, with the increase in BMP reaching 30% and 25% for M and T samples, respectively, (Table 2). Ball milling (VBM) did not improve methane production, as a slight decrease in BMP values was even observed in comparison to KM1 fractions. During the present study, and among samples with a similar composition, the BMP increase was not found to be related to the increase in soluble COD, which was enhanced by ball milling. In contrast, ultrafine milling with a shear mill led to a significant rise in the BMP of sample T, reaching 168 mL CH_4_/gVS, which is 53% and 71% higher than the BMP of KM1 and KM4 fractions, respectively.

In fact, the presence of lipids can affect mechanical dissociation and increase particle agglomeration with VBM. Recently, Williams et al. demonstrated how a higher lipid content significantly affects mechanical fractionation and dissociation and increases particle size by agglomeration [26]. The T biomass was submitted to two successive lipid extractions, which may have affected its matrix organization. This might also have affected their mechanical fractionation and dissociation, and as a consequence, increased holocellulose accessibility and methane production. Barakat et al. reported that chemical and physicochemical pretreatments increase mechanical dissociation, reduce energy consumption, decrease particle size and increase cellulose accessibility [14]. However, during the present study, these effects were not noticeable. The lipids contained in the M samples did not hinder milling, while the consumed energy was similar to that of the T sample milling.

Concomitantly, the methane yields of fractions obtained after different dry separation processes were investigated (Table 2). Maximum methane yields were obtained from the smallest sieving fractions SV < 0.9 of M and T biomasses, producing about 21% and 27% more methane than olive pomace ground at 1 mm (KM1) and 58% and 43% more than larger material (KM4), respectively. In contrast, the SV > 0.9 fractions produced 57% and 11% less methane than the material ground at 1 mm for M and T biomasses, respectively. This could be mainly explained by the higher lipid and protein content in SV < 0.9 fractions. The C/N ratio is known to be a key parameter in anaerobic digestion. Indeed, a low C/N ratio may lead to ammonium ion inhibition. Conversely, a high C/N ratio does not favor the development of cell biomass, since NH_4_^+^ is crucial for cell structure development and bacterial growth [27]. The C/N ratio is known to be optimal when ranging between 20 and 35. In the case of this study, the C/N of the SV > 0.9 fractions were 185 and 97 compared to 34 and 28 in the SV < 0.9 fractions from M and T olive pomaces, respectively (Table 1). The SV < 0.9 fraction C/N ratios are thus included within the optimal range. This result may be of interest for further full-scale applications.

BMP tests after electrostatic separation, pointed to slight differences between fractions (ESn− and ESp+). The positively charged fraction ESp+ produced 5% more methane than the UFM0.1 sample, while the negatively charged fraction ESn− produced 3% less methane than the UFM0.1. However, as previously reported in the literature, ESn− and ESp+ differ in their cellulose, protein and lignin composition. ES affects proteins and fibres contained in the ESn− and ESp+ fractions, due to their ability to charge either positively or negatively [22]. As a result, the ESn− presented a higher cellulose content (15%) than the ESp+ (9%) and UFM0.1 (11%). Nevertheless, its lignin content (31%) was lower than those of the ESp+ (41%) and UFM0.1 (31%). Barakat et al. investigated the coupling of ES and enzymatic hydrolysis of wheat straw [23]. After ES, the wheat straw fractions were hydrolyzed with an enzymatic cocktail. The positively charged fractions ESp+ had a maximum glucose yield of about 254 mg glucose g^−1^ compared to 130 mg glucose g^−1^ in the initial fraction. The authors suggested that ES technology might allow for a chemical enzyme-accessible biomass to be isolated, unlike a non-fractionated biomass.

Literature reports BMP values about 330 mL/gVS, 170–280 mL/gVS, and 220–260 mL/gVS, for traditional, two-phase and three phase process OP, respectively (Table S1). The olive pomaces used in this work produced less methane, especially for the T biomass. This may arise from the difference in the olive pomace chemical composition, which in turn depends on the geographical origins and types of oil extraction processes. Alkaline pretreatment proved to be effective in enhancing olive pomace hydrolysis, COD and polyphenol solubilization. However, at an optimized temperature and NaOH dose, the maximal methane obtained was 22% higher than the raw OP [28]. According to Rincon et al., ultrasonic pretreatment improved olive pomace solubilization, and increased methane production by 17% [29].

Pellera et al. reported that chemical pretreatments using ethanol, citric acid, and hydrogen peroxide were effective in solubilization of OP and enhancement of its methane production rate; however the cumulative methane produced was lower than in the untreated sample [11]. Donoso-Bravo et al. reported the effect of milling on the kinetic parameter values of raw and 3 mm milled samples. The maximum methane production rate was enhanced, although only a 5% methane yield increase was obtained [19]. Fenton reagent addition and steam explosion pretreatments had a negative impact on methane production, achieving 50% of reduction for steam explosion [19,30]. Indeed, high temperature and pressure conditions can lead to the formation of recalcitrant compounds, which affect the anaerobic digestion process [31]. However, after treatment at 148 °C, olive pomace produced 70% more methane than for the raw sample [19]. A similar increase in methane yield was observed in the current study with T olive pomace after ultrafine milling. Similarly, knife milling at 1 mm of M sample increased methane production by 30% compared to the control. It can thus be concluded that the results from this study are significant compared to literature. The dry milling and fractionation used here can be considered as environmentally friendly and efficient in methane potential enhancement.

### 2.3. Pretreatment Energy Efficiency

Table 3 presents the energy requirements of each device used. In the case of this study, the energy needed for heating and stirring the digester was not taken into account as it is common to all cases. Milling is one of the most energy consuming processes in industry. However, knife milling at 4 mm required the least amount of energy, i.e., only 0.0327 kWh/kg of olive pomace. This was followed by milling at 1 mm (0.27 kWh/kg) and finally vibro-ball milling. The latter was the most energy demanding, using about 19.75 kWh/kg, i.e., nine-fold the energy consumed by ultrafine knife milling (1.87 kWh/kg). As for sieving and electrostatic separation, the consumed energy was about 0.075 kWh/kg and 1.56 kWh/kg, respectively.

Mechanical pretreatment can enhance methane production; however, an energy balance should be made to evaluate its efficiency. In this study, ultrafine milling and electrostatic separation increased methane yields to more than 70%, although they also consume more energy than methane production can compensate for, especially when cogeneration performance with a power yield of about 40% is taken into account. Nevertheless, as the SV > 0.9 fractions for both M and T samples, as well as the negatively charged fraction, had lower BMP values, and resulted in less energy recovery, these fractions were not taken into consideration in calculations of efficiency.

The energy balance was positive after combining knife milling and sieving with anaerobic digestion. The maximum recovered energy was about 7.2 kJ/gVS after knife milling of the M sample to 1 mm. For the T sample, knife milling with a 4 mm screen was the most effective milling technique, producing 3.30 kJ/gVS after energy consumption had been taken into account. The energy balance after sieving was positive, but still lower than that of knife milling, obviously because the SV < 0.9 fractions only represented 27% and 26% of the initial amount of M and T samples, respectively.

To conclude, knife milling at four and 1 mm lead to improved methane production with less energy consumption. Although its BMP value is strongly enhanced, ultrafine knife milling cannot be efficient due to its high-energy requirement. Ball milling also utilized more energy than it produced. It is noteworthy that the efficiency of ball milling can be enhanced with water addition in order to save on energy consumption [32].

**Figure 5:**
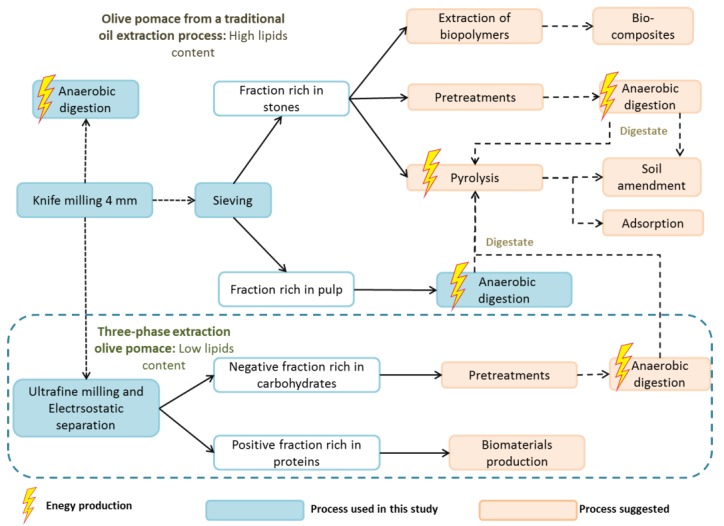
Valorisation routes suggested for all the studied fractions.

The SV > 0.9 fractions and both positive and negative fractions following electrostatic separation led to a negative energy balance. However, by taking into account their carbon and fiber composition, these fractions can be subjected to other valorization routes (Figure 5). The stone-rich fraction (SV > 0.9 presenting a recovery yield greater than 70%) can be used directly as an adsorbent [33] or subjected to pyrolysis for energy production [34]. It can also be used for bio-composite production [20,35]. Moreover, these types of fractions can be subjected to chemical or physical pretreatment prior to anaerobic digestion. This should increase their accessibility as well as enhance the degradation of the fibers they contain. Likewise, the negative fraction after electrostatic separation is rich in carbohydrates so it can be subjected to pretreatment before anaerobic digestion. Proteins can be extracted from the positive fraction, which can be used for the production of biomaterial.

Few published studies calculated the energy balance of physicochemical pretreatments for OP anaerobic digestion as reported in Table S1. Pretreatments such as OP sonication [29] and thermochemical pretreatments [11] were characterized by negative energy gains that are related to their high energy requirements. Conversely, as previously mentioned, mechanical pretreatments such as knife milling and sieving were more efficient, with a positive energy balance (Table S1).

## 3. Materials and Methods

### 3.1. Biomass and Inoculum

Two olive pomace (OP) biomasses were collected from a traditional cooperative in the Kalaa region in central Morocco (sample named M) and from a three-phase oil extraction industrial process company “Société générale des huileries du Sahel” in Sousse on the eastern coast of Tunisia (sample named T). The solid residues, containing fragments of stones, skin, seed, and pulp, were naturally sun-dried and then stored at ambient temperature. The inoculum used for BMP tests was an anaerobic sludge obtained from a mesophilic UASB reactor in Marseille dedicated to treating wastewater from a sugar factory. After reception, the inoculum was stored and stirred under mesophilic conditions (35 °C) and was periodically fed with ethanol to maintain methanogenic activity. Its volatile solids (VS) content was 40 g/L.

### 3.2. Mechanical Fractionation and Separation

**Figure 6:**
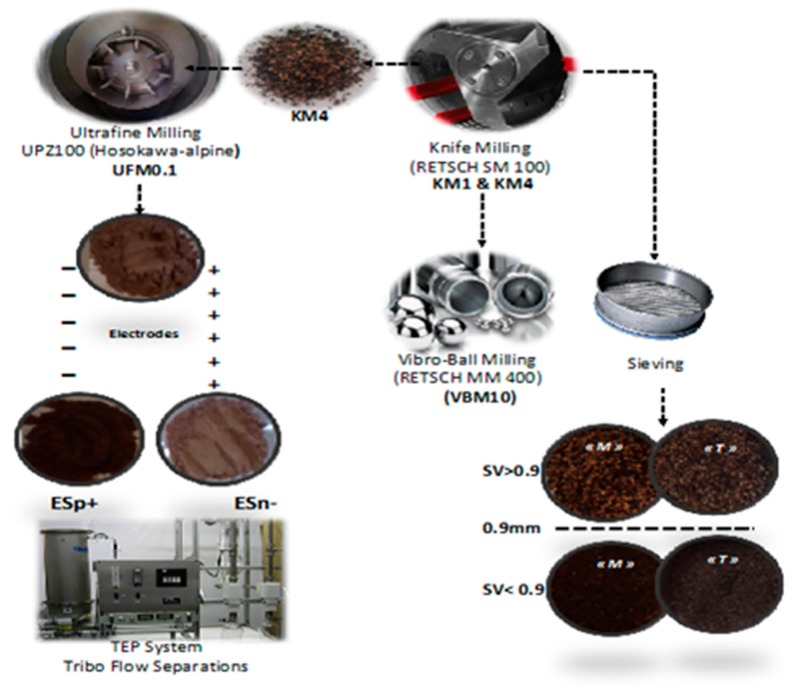
Mechanical pretreatments and fractions studied.

The OP biomasses were grinded by knife milling (RETSCH SM 100, Restch, Haan, Germany) using a 4 mm screen, as illustrated in Figure 6. The resulting (KM4) fraction was then subjected to knife milling using a 1 mm screen size, thus producing the (KM1) fraction. OP fraction (KM4) also underwent vibro-ball milling (VBM10) for 10 min (RETSCH MM 400). The OP ground fractions KM4 of T and M samples were then subjected to sieving with the vibratory sieve shaker (Analyset 3 Pro, Fritsch, Idar-Oberstein, Germany). Size distribution was determined by successive screening with 4, 2, 0.9, 0.5, and 0.25 mm screens. The 0.9 mm screen was used for sieving separation processes (SV < 0.9 and SV > 0.9). The KM4 of T sample was finely ground by impact and shear mill UPZ100 (Hosokawa-alpine, Augsburg, Germany) using a 0.1mm screen size (UFM0.1) operated at room temperature, at a speed of 18 000 rpm, and a feeder speed of 2 kg h^−1^. The (UFM0.1) fraction was subjected to an electrostatic separation process using (TEP System, Tribo Flow Separations, Lexington, KY, USA). Biomass M was not submitted to electrostatic separation as its high lipid content makes this process impossible to apply. The feeding system of the separator was operated at 150 rpm, and the OP (UFM0.1) particles were conveyed by compressed air into a charging line. The charged OP particles were then introduced into a separation chamber containing two high voltage electrodes (10000 V), where the positively charged particles (ESp+) were attracted by the negative electrode and the negatively charged particles (ESn−) were attracted by the positive electrode.

### 3.3. Biochemical Methane Potential (BMP) Tests

Biochemical methane potential (BMP) tests were performed to assess OP biodegradability. Batch BMP tests were carried out in triplicate in 500 mL flasks with 400 mL working volume. Both untreated and pretreated samples were added to the inoculum at a ratio of 1 gVSOP/gVS inoculum. Oligoelement, macroelement, and buffer solutions, at concentrations provided by Monlau et al. were added in volumes of 4, 25.8, and 20.8 mL, respectively [36]. The flasks were set to mesophilic conditions (35 °C) with continuous stirring. Blank controls with the same composition, but without substrate addition were made to measure the endogenous methane production from inoculum, which was subtracted from OP methane productions. The endogenous production ranged between 10 and 15% of total methane production. The calculation of the volume of produced methane was based on pressure measurements, according to the ideal gas law and on the biogas composition obtained using gas chromatography GC CLARUS 480-Perkin Elmer, as described in Sambusiti et al. [37]. The latter was equipped with two columns: A RtUBond column for separating CO_2_ and H_2_S from the other gases, which in turn could be separated with a RtMolsieve column with helium as carrier gas. Column temperature was 65 °C, while the injector and thermal conductivity detector were set at 200 °C.

The theoretical methane potential formula based on the organic fraction of feedstock is given as follows [38]:

(1) BMPth=415×%carbohydrates+496×%proteins+1014×%lipids

### 3.4. Analysis

Assuming that grinding does not affect the composition of OP and to reduce their heterogeneity, all samples underwent ball milling for cellulose, hemicellulose, and lignin, protein, lipid, volatile solid (VS), and total solid (TS) analysis. All measurements were performed in triplicate. The measurement of TS and VS contents was performed using the APHA (American Public Health Association) method. Hemicellulose, cellulose and lignin contents were determined according to Monlau et al. After acid hydrolysis with sulfuric acid at 72%, sugars were analyzed by HPLC. The lignin content was then determined by subtracting the ash content from the remaining residue [39]. For lipid and CHNS analysis, thinly milled samples were used, VBM10 for M biomass and UFM0.1 for T biomass. Lipids were extracted using the spectrophotometric sulfo-phospho-vanillin method [40]. The CHNS content was measured by elemental analysis using Thermo Scientific FlashSmart analyzer, via flash combustion at 950 °C. The protein content was then obtained by multiplying the N content by 6.25 [41]. Sample solubilization following pretreatment was determined according to the soluble chemical oxygen demand (COD) and polyphenol measurements, using the same water to solid ratio as in the preparation of BMP tests. The amount of soluble COD was evaluated after maceration of 2 g of OP for 16 h, in 400 mL of Milli-Q water with stirring at 100 rpm and under ambient temperature. The mixture was then diluted with Milli-Q water before being transferred to the Spectroquant test kits and the COD value was read by a HACH DR/2000 spectrophotometer at 620 nm. The soluble polyphenol content was measured using a spectrophotometric method. 100 µL of the total liquid fraction were added to 500 µL of a 10 fold diluted Folin-Ciocalteu solution, and 400 µL of a Na_2_CO_3_ solution at 75 g/L. The mixture was stirred in a vortex mixer, and left in a bath at 40 °C for 5 min; it was then analyzed in triplicate with a spectrophotometer at a wavelength of 735 nm using gallic acid as a reference. Polyphenol concentrations were then expressed by grams of gallic acid equivalent per grams of volatile solids.

The particle size distribution of the samples was determined by laser diffraction using a Mastersizer 2000 in combination with the Scirocco 2000 (Malvern Instruments, Worcestershire, UK). Results were expressed in terms of D50, which represents the diameter at which 50% of the sample is composed of particles with a diameter smaller and bigger than this value. The color of the fractions obtained by electrostatic separation and sieving was measured with a colorimeter (Minolta CR-410 Colorimeter, Nieuwegein, Netherlands), using the L.a.b. colour system. The L* value characterizes the luminance, while a* and b* values are indicators of sample color (a: from green to red, and b: from dark blue to yellow). Measurements were performed in duplicate, and the averages of L*, a*, and b* were calculated. The total color difference is given as follows:

(2) $$\Delta E^{*}={\left[\Delta L^{*2}+\Delta a^{*2}+\Delta b^{*2}\right]}^{1/2}$$

### 3.5. Specific Energy Measurement

The power consumed during different pretreatments was measured with a wattmeter [14] and specific energy was calculated as follows:

(3) $$E_{M}=\frac{\int_{0}^{t}\left(P_{t}-P_{0}\right)dt}{m}=\frac{\int_{0}^{t}\Delta P_{t}dt}{m}$$

With *Pt* the power (watt) consumed at a time *t* (s), *P*_0_ the power consumption (watt) under idle conditions (without biomass), and m the mass (kg) of biomass to be ground.

For separation techniques, prior milling is required. The energy formula becomes:

(4) $$E_{t}=E_{sep}+E_{M}$$

Energy efficiency is defined by the difference between the methane energy produced after a pretreatment and the energy consumed. In this study, pretreatments are compared to knife milling at 4 mm. The efficiency or energy balance is given as follows:

(5) $$E_{f}=E_{Out}-E_{In}$$

With, *E_out_* (kJ CH_4_/gVS) the methane energy produced with a higher heat value of methane estimated at 55.6 MJ/kg [42] and *E_in_* (kJ/gVS) the energy consumed during pretreatment

## 4. Conclusions

The separation techniques affected the distribution of carbohydrates, proteins, and lipids in the different fractions, while milling influenced the bioavailability and the capacity to solubilize organic compounds. The maximal methane potential was generally related to maximal lipid and protein contents as well as maximal solubilization. For knife milling and sieving, anaerobic digestion can compensate for the energy needed for pretreatment without taking into account the requirements for digester heating and stirring. Best energy efficiency was observed after knife milling, while ball milling and ultrafine milling were the most energy demanding. However, it remains crucial to regard the energy efficiency results based on BMP tests as a preliminary evaluation. Using this approach, pretreatments that should be assessed in combination with continuous anaerobic digestion reactors can be selected. Indeed, energy balance evaluation in pilot continuous systems will be required before any further scale-up of the process. Finally, this study has been carried out with a lignin-rich substrate. Therefore, the results from milling or fractionation pretreatments and energetic efficiency might be different with substrates that have lower lignin content and which would thus be more favourable towards anaerobic digestion processes.

## Figures and Tables

**Table 1:** Properties of different olive pomaces and their fractions.

Sample	M			T					
	VBM10	SV < 0.9	SV > 0.9	VBM10	SV < 0.9	SV > 0.9	UFM0.1	ESn−	ESp+
Yield (%)	100	27 ± 1	73 ± 1	100	26 ± 2	74 ± 2	100	65 ± 5	35 ± 3
TS (% Wb)	94 ± 2	94 ± 2	92 ± 2	91 ± 0.2	92 ± 0.1	92 ± 0.5	91 ± 0.0	91 ± 0.0	91 ± 0.0
VS (% Wb)	82 ± 3	83 ± 2	81 ± 2	80 ± 2	87 ± 3	81 ± 2	82 ± 2	80 ± 1	87 ± 2
D_50_ (µm)	113 ± 5	507 ± 22	-	210 ± 4	601 ± 17	-	54 ± 2	49 ± 2	75 ± 3
**Elemental Analysis**									
C (%)	48 ± 0.1	51 ± 0.1	48 ± 0.1	48 ± 0.1	49 ± 0.1	48 ± 0.1	48 ± 1	-	-
H (%)	7 ± 0.1	7 ± 0.2	6 ± 0.3	7 ± 0.01	6 ± 0.1	6 ± 0.3	7 ± 0.01	-	-
N (%)	0.5 ± 0.02	1.5 ± 0.06	0.3 ± 0.09	0.6 ± 0.04	2 ± 0.004	0.5 ± 0.1	0.6 ± 0.04	0.5 ± 0.03	1.4 ± 0.1
S (%)	0.1 ± 0.002	0.2 ± 0.03	0.04 ± 0.002	0.05 ± 0.005	0.2 ± 0.02	0.1 ± 0.004	0.05 ± 0.005	-	-
Hemicellulose(% Db)	11 ± 0.2	4 ± 0.4	13 ± 0.3	16 ± 0.3	11 ± 1	17 ± 0.3	17 ± 1	20 ± 1	11 ± 0.4
Cellulose(% Db)	11 ± 0.1	5 ± 0.2	14 ± 1	10 ± 2	10 ± 1	11 ± 1	11 ± 1	15 ± 1	9 ± 0.4
Lignin (% Db)	37 ± 2	40 ± 1	30 ± 0.4	31 ± 0.03	34 ± 3	30 ± 4	36 ± 2	31 ± 2	41 ± 3
Lipids (%Wb)	14 ± 0.4	30 ± 0.8	9 ± 0.2	6 ± 0.2	6 ± 0.2	3 ± 0.1	6 ± 0.2	-	-
Proteins (% Wb)	3 ± 0.1	9 ± 0.4	2 ± 0.6	3 ± 0.2	11 ± 0.02	3 ± 0.6	3 ± 0.2	3.1 ± 0.2	9 ± 0.7
TMY ^1^ (mL/gVS)	249	386	213	168	203	161	191	-	-
**Colour**									
a*	8 ± 0.3	8 ± 0.3	10 ± 0.1	8 ± 0.6	7 ± 0.3	8 ± 0.1	5 ± 0.4	5 ± 0.3	6 ± 0.4
b*	9 ± 0.6	19 ± 1	18 ± 0.3	16 ± 1	12 ± 1	16 ± 1	13 ± 1	17 ± 1	12 ± 1
L*	33 ± 1	28 ± 1	34.5 ± 0.5	47 ± 4	37 ± 1	44 ± 1	53 ± 2	63 ± 2	43 ± 1
∆E*	-	5	9	-	11	3	-	10	6

^1^ TMY (Theoretical methane yield).

**Table 2:** Methane production and solubilization results.

Samples		sCOD (mg/g_VS_)	sCOD (mg/L)	sPolyphenols (mg GAE/g_VS_)	EMY ^1^ (mL CH_4_/g_VS_)	EMY/TMY ^2^ (%)	Enhancement in BMP (% KM4)	Enhancement in BMP (% KM1)	Enhancement in BMP (% UFM0.1)
**M**	KM4	151 ± 4	604 ± 16	7.4 ± 0.1	188 ± 18	76	-		
	KM1	159 ± 2	636 ± 8	7.5 ± 0.2	246 ± 10	98	+30		
	VBM10	263 ± 1	1052 ± 4	12.1 ± 0.2	224 ± 7	90	+19	−9	
	SV < 0.9	245 ± 1	980 ± 4	10.6 ± 0.2	298 ± 7	77	+58	+21	
	SV > 0.9	121 ± 1	484 ± 4	5.2 ± 0.2	105 ± 12	49	−44	−57	
**T**	KM4	115 ± 4	460 ± 16	4.2 ± 0.1	98 ± 14	58	-		
	KM1	156 ± 3	624 ± 12	5.3 ± 0.3	110 ± 5	65	+12		
	VBM10	162 ± 3	648 ± 12	7.7 ± 0.2	108 ± 15	64	+10	−2	
	UFM0.1	197 ± 4	788 ± 16	12.0 ± 0.2	168 ± 8	88	+71	+53	
	SV < 0.9	205 ± 7	820 ± 28	7.5 ± 1.8	140 ± 4	69	+43	+27	
	SV > 0.9	140 ± 3	560 ± 12	6.4 ± 1.2	98 ± 7	61	0	−11	
	Esn−	182 ± 5	728 ± 20	7.7 ± 0.2	163 ± 10	-	+66	+48	−3
	Esp+	223 ± 5	892 ± 20	13.8 ± 0.4	177 ± 18	-	+81	+61	+5

^1^ EMY (Experimental methane yield). ^2^ TMY (Theoretical methane yield).

**Table 3:** Energy balance and dry fractionation efficiency.

Pretreatments	KM4		KM1		VBM10		UFM0.1	Sieving (SV < 0.9)		ES
Sample	M	T	M	T	M	T	T	M	T	T Esp+
Energy consumed by each process (kWh/kg OP)	0.03 ± 0.01		0.27 ± 0.09		19.75 ± 3.17		1.87 ± 0.12	0.075 ± 0.00		1.56 ± 0.11
Total energy consumed (kWh/kg OP)	0.03		0.30		19.78		1.90	0.11		3.46
Energy input (kJ/gVS init *)	0.14	0.15	1.32	1.36	86.53	88.91	8.56	0.48	0.48	15.58
CH_4_ (mL/gVS **)	188 ± 18	98 ± 2	246 ± 10	110 ± 5	224 ± 7	99 ± 2	168 ± 8	298 ± 7	140 ± 4	177 ± 17
**Amount Recovered in the Fraction (%)**								27	26	35
Energy output (kJ/gVS init)	6.5	3.4	8.5	3.8	6.1	3.4	5.8	2.8	1.2	2.2
Energy balance (kJ CH_4_/gVS init)	+6.36	+3.30	+7.2	+2.4	−80.4	−85.5	−2.8	+2.32	+0.72	−13.7

* VS init of olive pomace before fractionation; ** VS of olive pomace after fractionation.

## Supplementary Materials

The following are available online, Table S1: Pretreatments of OP in the literature and in the present study.
