# Supplementary material for: Combination of Dry Milling and Separation Processes with Anaerobic Digestion of Olive Mill Solid Waste: Methane Production and Energy Efficiency
Source: Molecules. 2018 Dec 12;23(12):3295. doi: 10.3390/molecules23123295 (PMC6321404; doi:10.3390/molecules23123295)
Supplement: Supplementary file 1 [file molecules-23-03295-s001.pdf]

Supplementary material

Combination of Dry Milling and Separation Processes with Anaerobic Digestion of Olive Mill Solid Waste: Methane Production and Energy Efficiency

Doha Elalami [1,2,3], Hélène Carrère [1,*], Karima Abdelouahdi [2], Abdallah Oukarroum [3], Driss Dhiba [3], Mohamed Arji [4] and Abdellatif Barakat [5]

1 LBE, Université de Montpellier, INRA, 102 Avenue des Etangs, F-11100 Narbonne, France; Doha.ELALAMI@um6p.ma
2 LCME, FST Marrakech, Université Cadi Ayyad, Marrakech, 40000 Morocco; abdelouahdi@gmail.com
3 Mohammed VI Polytechnic University (UM6P), 43150 Benguerir, Morocco; Abdallah.OUKARROUM@um6p.ma (A.O.); D.DHIBA@ocpgroup.ma (D.D.)
4 OCP Group, Complexe industriel Jorf Lasfar, BP 118 El Jadida, Morocco; mohamed.arji@ocpgroup.ma
5 IATE, CIRAD, Montpellier SupAgro, INRA, Université de Montpellier, 34060 Montpellier, France; abdellatif.barakat@inra.fr
* Correspondence: helene.carrere@sinra.fr; Tel.: +33-468-425-168

Table S1. Pretreatments of OP in the literature and in the present study.

Pretreatment	Conditions	Methane before pretreatment (ml/gVS)	Methane after pretreatment (ml/gVS)	Methane enhancement (%)	Energy balance (kJ/gVS)	Ref
Thermo-alkaline	Three-phase OP NaOH (4% VS) 90°C, 4 h	221.5	273.0	+22%	Nd	[28]
Fenton reagent addition	Traditional extraction OP H2O2/[Fe2+] = 1000, [Fe2+] = 1.5 mM, 120 min, pH 3	335.5	262.1	-22%	Nd	[30]
Thermal and Steam explosion	Two-phase OP P= 12 bar 166°C, 30 min	255.6	119.4	-50 %	Nd	[19]
Thermal	Two-phase OP 148°C, 30 min	255.6	434.2	+70 %	Nd	
	Two-phase OP 180°C, 180 min	373	380	+2%	Nd	[9]
Ultrasonic	Two-phase OP 200W, 24 kHz 90 min	373	393	+5%	−192.3	[29]
Milling	Two-phase OP Manual mill to 3mm	170	176	+4%	Nd	[19]
Thermochemical pretreatment	Three-phase OP Citric acid at 90°C, 4 hours 1mmol/gVSsubstrate	258.7	217.3	-16%	-111.7	[11]
	H2O2 at 90°C for 4 hours 1mmol/gVSsubstrate	258.7	176.3	-47%	-113.2	
	Ethanol at 90°C for 4hours 1mmol/gVSsubstrate	258.7	197.8	-23	-112.4	
Milling	Traditional extraction OP	188	246	+31%	+7.2	Present study

	Knife milling 1 mm				
	Three-phase OP Vibro-ball milling, 10 min	98	108	+10	-85.5
	Three-phase OP Ultrafine milling 0.1 mm	98	168	+71%	-2.8

Nd Not determined.
